# Assessment of flow within developing chicken vasculature and biofabricated vascularized tissues using multimodal imaging techniques

**DOI:** 10.1038/s41598-021-97008-w

**Published:** 2021-09-14

**Authors:** Prasanna Padmanaban, Ata Chizari, Tom Knop, Jiena Zhang, Vasileios D. Trikalitis, Bart Koopman, Wiendelt Steenbergen, Jeroen Rouwkema

**Affiliations:** 1 Vascularization Lab, Department of Biomechanical Engineering, Technical Medical Centre, Faculty of Engineering Technology, University of Twente, 7500 AE Enschede, The Netherlands; 2 Biomedical Photonic Imaging, Technical Medical Centre, Faculty of Science and Technology, University of Twente, 7500 AE Enschede, The Netherlands

**Keywords:** Biological models, Imaging, Microscopy, Medical research, Applied optics, Lasers, LEDs and light sources, Optical techniques

## Abstract

Fluid flow shear stresses are strong regulators for directing the organization of vascular networks. Knowledge of structural and flow dynamics information within complex vasculature is essential for tuning the vascular organization within engineered tissues, by manipulating flows. However, reported investigations of vascular organization and their associated flow dynamics within complex vasculature over time are limited, due to limitations in the available physiological pre-clinical models, and the optical inaccessibility and aseptic nature of these models. Here, we developed laser speckle contrast imaging (LSCI) and side-stream dark field microscopy (SDF) systems to map the vascular organization, spatio-temporal blood flow fluctuations as well as erythrocytes movements within individual blood vessels of developing chick embryo, cultured within an artificial eggshell system. By combining imaging data and computational simulations, we estimated fluid flow shear stresses within multiscale vasculature of varying complexity. Furthermore, we demonstrated the LSCI compatibility with bioengineered perfusable muscle tissue constructs, fabricated via molding techniques. The presented application of LSCI and SDF on perfusable tissues enables us to study the flow perfusion effects in a non-invasive fashion. The gained knowledge can help to use fluid perfusion in order to tune and control multiscale vascular organization within engineered tissues.

## Introduction

The inclusion of multiscale vascular networks portraying a correct hierarchical organization within engineered tissues is essential for the tissue viability and function. Vascular networks that include large vessels and small capillaries are essential for transporting oxygen and nutrients to allow for tissue survival. Large vessels are needed to bridge distances without large pressure drops, whereas small capillaries are needed to access all cells within the tissue. Even though the field of biofabrication has progressed fast over the past years, fabricating a large tissue construct with a resolution down to the smallest capillaries (~ 5 μm) remains challenging. Therefore, a stable hierarchically organized vascular network including all relevant size scales, will likely still rely on tissue remodeling. As fluid flow shear stresses is one of the key regulators of vascular organization and remodeling^1,2^, it is important to understand the blood flow within developing vascular networks of varying complexity.

For a long time, vascular network formation during embryogenesis has been considered to be genetically predetermined^3^. However, more recent studies showed that vascular cells can display plasticity with respect to local cues such as hemodynamics, especially blood flow shear stresses, meaning that the organization is influenced by the environment^4^. Hemodynamics-driven vascular organization is vividly observable in the chick embryo where the arteries and veins appear just a few hours after the onset of the heartbeat and subsequently blood perfusion. Perturbation of blood flow within the naturally formed vascular network of the chick embryo has been used to observe the effect of hemodynamic changes on vascular organization^4–6^. Additionally, in vitro studies have shown that mechanical signals such as fluid flows^7,8^ and wall shear stresses^9,10^ play an important role in regulating the different stages of vascular organization. However, even though the importance of fluid flow related mechanical signals in a wide range of in vitro cellular organizational phenomena has been shown, it has not been possible so far to completely elucidate the physiological significance of these mechanical signals, largely due to a lack of accessible angiogenesis models and integrated imaging platforms fit for long-term culture.

Due to this, accessible angiogenesis models have become a key focus in tissue engineering. Multiple in vivo models exist for studying the vascular responses to biomaterials and drug testing. Examples include zebra fish^11–13^, mice, skin flap windows^14,15^ and snake embryos^16,17^. These models are generally complex and are associated with ethical concerns. Moreover, these models provide only a small area for imaging and often biomaterials/drugs are tested at random locations, lacking spatiotemporal control. Due to these limitations, the shell-less ex-ovo culture of chick embryo and its chorioallantoic membrane (CAM) has become a popular model for studying vascular network organization^18–21^.

Optical methods used for imaging flow within vascular networks include optical coherence tomography (OCT)^22^, photoacoustics^23^, ultrasound^24^, bioluminescence^25^ etc. OCT and photoacoustics provide three-dimensional images, however they suffer from limited field-of-view (FOV) and need a complicated experimental setup. To overcome these challenges, this study adopts laser speckle contrast imaging (LSCI)^26–28^ and side-stream dark field (SDF)^29^ microscopy to probe the spatial and temporal profile of blood flow distribution and erythrocyte velocities within individual capillaries. LSCI is noninvasive, requires a rather simple experimental setup and provides a wide FOV typically in the range of several square centimeters; however, it is a two-dimensional imaging modality. SDF also represents a simple, portable experimental setup with high sensitivity that provides fine, well-defined video recordings of capillary structures. Moreover, this modality uses light-emitting diodes (LED) as a light source rather than lasers as used in LSCI. The downside of SDF is that the probe covered by a disposable cap should touch the sample surface during imaging, which can result in perturbations of the developing tissue and causes concerns regarding the aseptic nature of cell and tissue culture.

**Figure 1:**
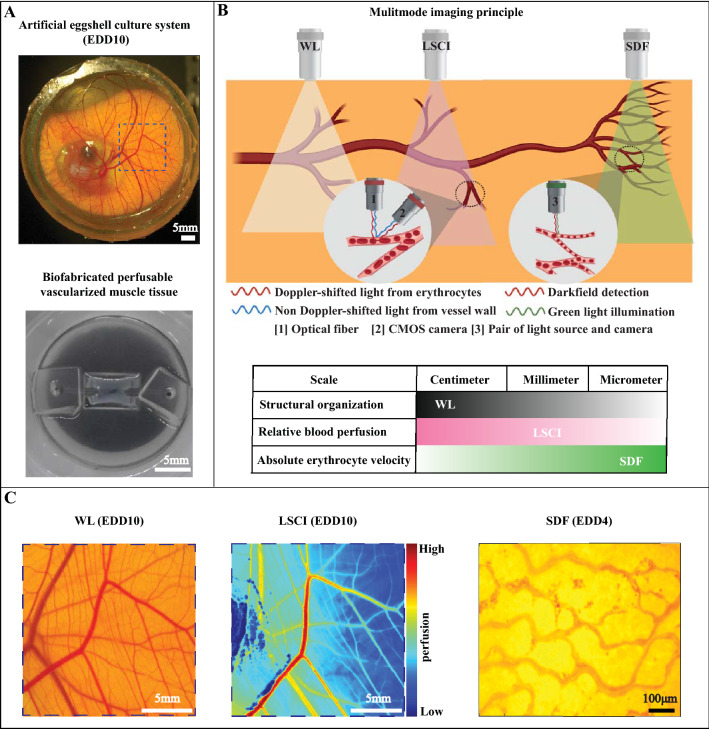
**Multimode imaging for probing vascular organization and associated flow dynamics.** Panel (**A**) shows the artificial eggshell culture system with fully developed chick embryo of embryo development stage day 10 exhibiting multiscale vasculature of varying complexity, as well as a biofabricated vascularized tissue analogue. Both systems are compatible with WL, LSCI and SDF imaging methods and represent the proof-of-concept application towards engineering functional tissue constructs. Panel (**B**) shows the schematics explaining the principle of multiple imaging methods used in this study, with highlighted potential and limitations of the above-mentioned imaging methods. Panel (**C**) represents the examples of output results obtained from multiple imaging methods. Abbreviations: WL—white light (color) imaging; LSCI—laser speckle contrast imaging and SDF—side-stream dark field microscopy; EDD—embryo development day. Panel (**B**) schematic figure was created using Biorender.com.

In this study, we prepared an artificial eggshell in which chick embryos were cultured from day 3 to day 10 of development. The complete vasculature was imaged using color imaging and LSCI. The former was used to quantify vessel properties such as diameter while the later was used to explore the blood flow level of the vasculature at different locations and times. Additionally, LSCI experiments were performed on biofabricated muscle tissues containing a perfusable channel as a proof-of-concept (POC) to show the application of LSCI in engineered tissues. An SDF probe was used to visualize capillary structures and erythrocyte velocity on several locations of the vasculature. Figure 1 gives an overview of the three imaging modalities. To get a more quantitative understanding of the LSCI data, flow-phantom experiments were carried out on microtubing of diameters ranging from 75 to 500 µm through which a blood-mimicking scattering fluid (Intralipid with dye) was pumped. The microtubing diameters were comparable with diameters of the vasculatures imaged in in vivo experiments. The microtubing was mounted on both static scattering and static absorbing media to study their influence on the LSCI measurements. Additionally, computational fluid dynamics simulations (velocity-driven model) were performed to estimate shear stresses within the multiple vessel diameters.

## Results

### Quantification of vessel properties within developing chick vasculature using color imaging

**Figure 2:**
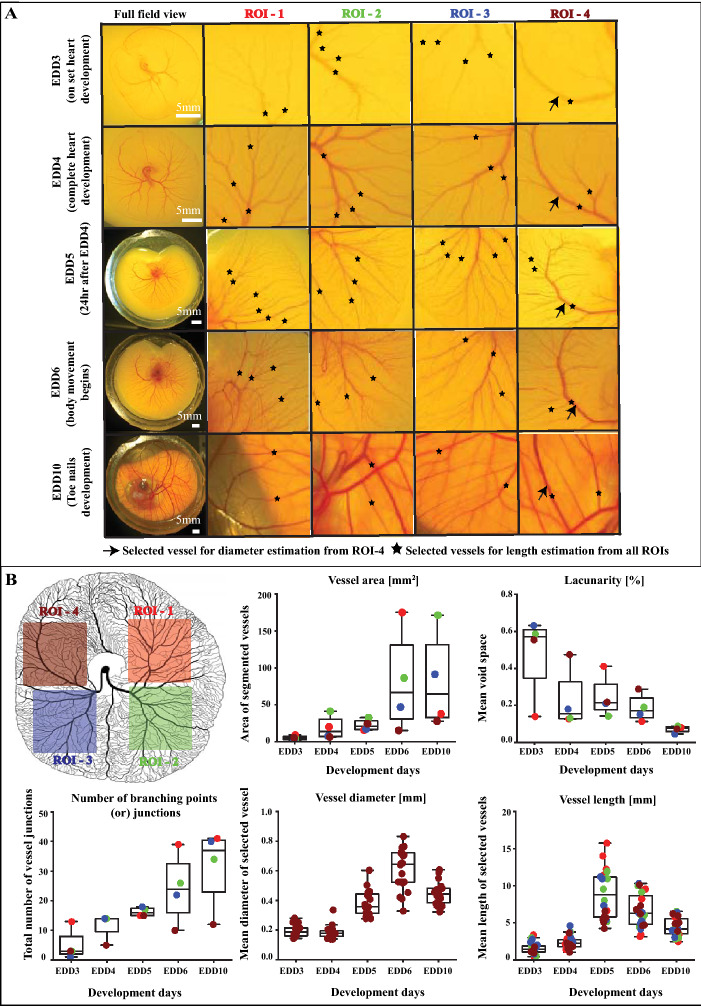
**Organization of developing vascular networks.** Panel (**A**) shows the snapshots of entire vascular networks of developing chick embryo including the CAM cultured within artificial eggshell culture system. The figures represent the developmental stages of day 3–6 and day 10, displaying the evolution of vascular network organization in both space and time. For videos, see Supplementary Video S6. Panel (**B**) represents the schematic figure of chick vascular network of the CAM development day 5 with highlighted ROIs, which are used for reference in Panel (**A**) and for the quantitative analysis. These multiple ROIs can be used for performing statistical analysis. Measured values are calculated from one sample (*n* = 1) and represented as mean ± SD along with individual data points. Measured values are compared within 4 different ROIs for different development days as indicated. Vessel diameter are calculated from single vessel of ROI-4 for different development days (highlighted with black arrows) and vessel length are calculated from multiple vessels from all ROIs (highlighted with black asterisks). For detailed explanation about quantitative analysis, see methods section and Supplementary Figure S2. Schematic figure in Panel (**B**) was created using Adobe Illustrator.

Figure 2A illustrates color images of the entire vasculature during 5 development days, namely 3–6 and 10. Since the culture system allows for a wide view of the vasculature from the top side, multiple ROIs are selected in order to perform statistical analysis (See Fig. 2B). The quantification metrics were performed as explained in methods section and Supplementary Figure S2. The results show an increasing trend in the vasculature area and number of branching points over time. The mean lacunarity (i.e. the void spaces in the image) were decreased over time, due to the increasing vascular sprouting and emergence of new vascular structures. An interesting trend was observed for the vessel diameter. Between EDD3 and EDD6, the average vessel diameter increased, which was accompanied by a wider spread in the vessel diameters. This points to a remodeling vascular network that becomes more multi-scale over time. On EDD10, the average vessel diameter was slightly decreased again, which may indicate a further stabilization of the formed vascular network. This is corroborated by the data on the average vessel length, which decreased between EDD5 and EDD10.

### Laser speckle contrast perfusion imaging

#### Validation of perfusion using microtubing flow phantoms

**Figure 3:**
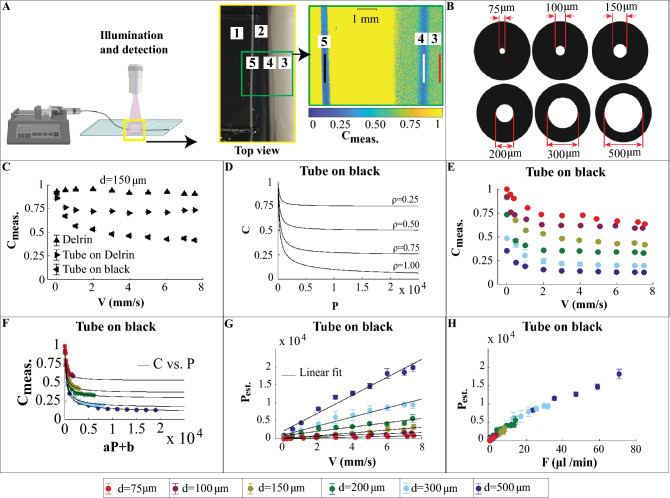
**LSCI on microtubing flow phantoms.** Panel (**A**) Left, schematic of syringe pump connected to a microtubing for Intralipid-dye perfusion and laser speckle contrast measurement. Middle, top view of the area for imaging. 1, glass plate mounted on microtubing and static media. 2, light isolating wall. 3, Delrin. 4, tube on Delrin. 5, tube on black. Right, representative speckle contrast map for the channel of *d* = 300 μm at *F* = 17 μl/min. The red, white and black ROIs indicate the regions in which $C_{{{\text{meas}}}}$ are calculated, respectively. The schematic figure in Panel (**A**) is created using Biorender.com. Panel (**B**) shows the microtubings of different diameters, representing the blood vessel diameter variation of the CAM. Panel (**C**) shows the measured speckle contrast (calculated based on Eq. (1), SI) versus the volumetric flux (calculated based on Eq. (4), SI) for the representative tube diameter of 150 μm as a comparison between the cases of Delrin, tube on Delrin and tube on black, respectively. Data points are mean ± standard deviation. Panel (**D**) illustrates relation between the speckle contrast and the perfusion shown in Eqs. (3) and (5), SI for various scattering ratios. Panels (**E**–**H**) correspond to the case of tube on black and the colored data points represent the different vessel diameters as stated in Panel (**H**). Panel (**E**) illustrates the measured speckle contrast versus the volumetric flux. Panel (**F**) illustrates the measured speckle contrast versus the shifted and scaled versions of the perfusion for each tube diameter overlapped with the theoretical curves of the speckle contrast versus perfusion. Panel (**G**) illustrates the estimated perfusion versus the volumetric flux with a linear fit for each tube diameter. Panel (**H**) is a plot of the estimated perfusion versus the flow rate.

The schematic diagram of the flow phantom study is depicted in Fig. 3A. For a comparison between the inner and outer diameters of the microtubes see Fig. 3B that visualizes the cross sections. For the experiments on each tubing, three regions were selected namely, Delrin, tube on black and tube on Delrin. The mean intensity of these areas detected on the camera sensor is shown in Supplementary Figure S4(A–C), respectively.

The measured speckle contrast versus the set volumetric flux on these regions for the representative tube diameter of 150 μm is depicted in Fig. 3C. It is observed that the measured speckle contrast on Delrin remains constant independent of the applied volumetric flux, which means that it acts as a baseline signal validating the stability of the light source during the measurement. For all volumetric fluxes, the drop in the measured speckle contrast for the case of tube on black was higher than the case of tube on Delrin. This observation is the case for all of the tube diameters used in this study (see Supplementary Figure S4(D-H)). For the case of tube on Delrin, the observed (backscattered) light includes contribution of light waves that (1) only propagate through the flow (experience Doppler shift); (2) only propagate through Delrin (static medium, no Doppler shift); (3) propagate through both the flow and Delrin. The cases (1) and (3) form dynamic speckle patterns of limited correlation time and the case (2) forms static speckle pattern of long correlation time (unity contrast). The speckle pattern is observed on the camera will be the result of superposition of both Doppler shifted and non-Doppler shifted light. However, for the case of tube on black, there is no contribution of light waves that propagate through a static background (Delrin) and in an ideal case (no static scattering material) fully dynamic speckle patterns are observed. Therefore, due to the presence of a partly static speckle pattern in the case of tube on Delrin, the observed speckle patterns will be of higher contrast (less blurred) than the case of tube on black. For a visualization of this situation see Supplementary Video S7 in which black and white regions correspond to tube on black and tube on Delrin, respectively. In Fig. 3E the influence of tube diameter is probed for the case of tube on black. By increasing the volumetric flux, the measured speckle contrast decreases. For a certain volumetric flux, increasing tube diameter causes a higher drop in the speckle contrast. Similar behavior is observed for the case of tube on Delrin (see Supplementary Figure S4(I)). The underlying reason can be explained by considering intensity fluctuations at each camera pixel within the exposure time. The frequency of the observed intensity fluctuations obeys the optical Doppler effect^30^. The larger the tube diameter, the more the probability of multiple Doppler scattering. A higher Doppler shift causes more fluctuations within the exposure time and therefore more blurred speckle patterns of lower contrast.

The theoretical model introduced in Eqs. (3) and (5), Supplementary Information SI estimates perfusion (effective velocity) as a function of exposure time and scattering ratio parameter *ρ*. Figure 3D shows the curves of speckle contrast versus perfusion for different values of scattering ratio parameter. For a certain perfusion value, a higher scattering ratio corresponds to a lower speckle contrast. The tube material, inner diameter, thickness and scattering background influence the scattering ratio parameter. To account for these effects and as explained in Supplementary Information SI, the scattering ratio parameter for each tube diameter and scattering background was chosen such that the estimated perfusion responded linearly to the applied flow.

As a demonstration of the fitting of the measured data with the theory shown in Fig. 3D, the speckle contrast was plotted versus perfusion according to the scattering ratio parameter for each tube diameter shown in Supplementary Table S3. Then, the measured speckle contrast data points were plotted versus a shifted and scaled version of the perfusion such that they matched with the theoretical curves. Figure 3F shows the result of the fitting for the case of tube on black. For the case of tube on Delrin see Supplementary Figure S4(J). The purpose of this fitting is to show that the measured data points match the behavior of the theoretical model, which is a way of validating the proper choice of the scattering ratio parameters. However, we acknowledge that the scaling and shifting parameters may not have a straightforward relation with the physical aspects such as speckle size and imaging system.

The estimated perfusion versus the applied volumetric flux for various tube diameters and the case of tube on black is shown in Fig. 3G. Although the estimated perfusion, to a good extent, is proportional to the applied volumetric flux, the slopes of the fitted lines depend on the tube diameter. Similar results are obtained for the case of tube on Delrin (see Supplementary Figure S4(K)). Notice that the estimated perfusion is a direct interpolation of the measured speckle contrast according to Eqs. (3) and (5), Supplementary Information SI; therefore, no scaling and shifting is applied. The estimated perfusion versus the applied flow rate for the case of tube on black is depicted in Fig. 3H. Now, as well as the proportionality, the estimated perfusion shows independency to the tube diameter, when plotted versus the applied flow rate. Similar results are achieved for the case of tube on Delrin (see Supplementary Figure S4(L)). Our analysis suggests that knowledge about the tube diameter is crucial in making perfusion estimation that is proportional to the applied volumetric flux or flow rate. Moreover, vessel diameter is an important geometrical parameter that constantly changes over the development and varies within the chick vascular system.

#### Blood flow distribution within vasculature of varying complexity

**Figure 4:**
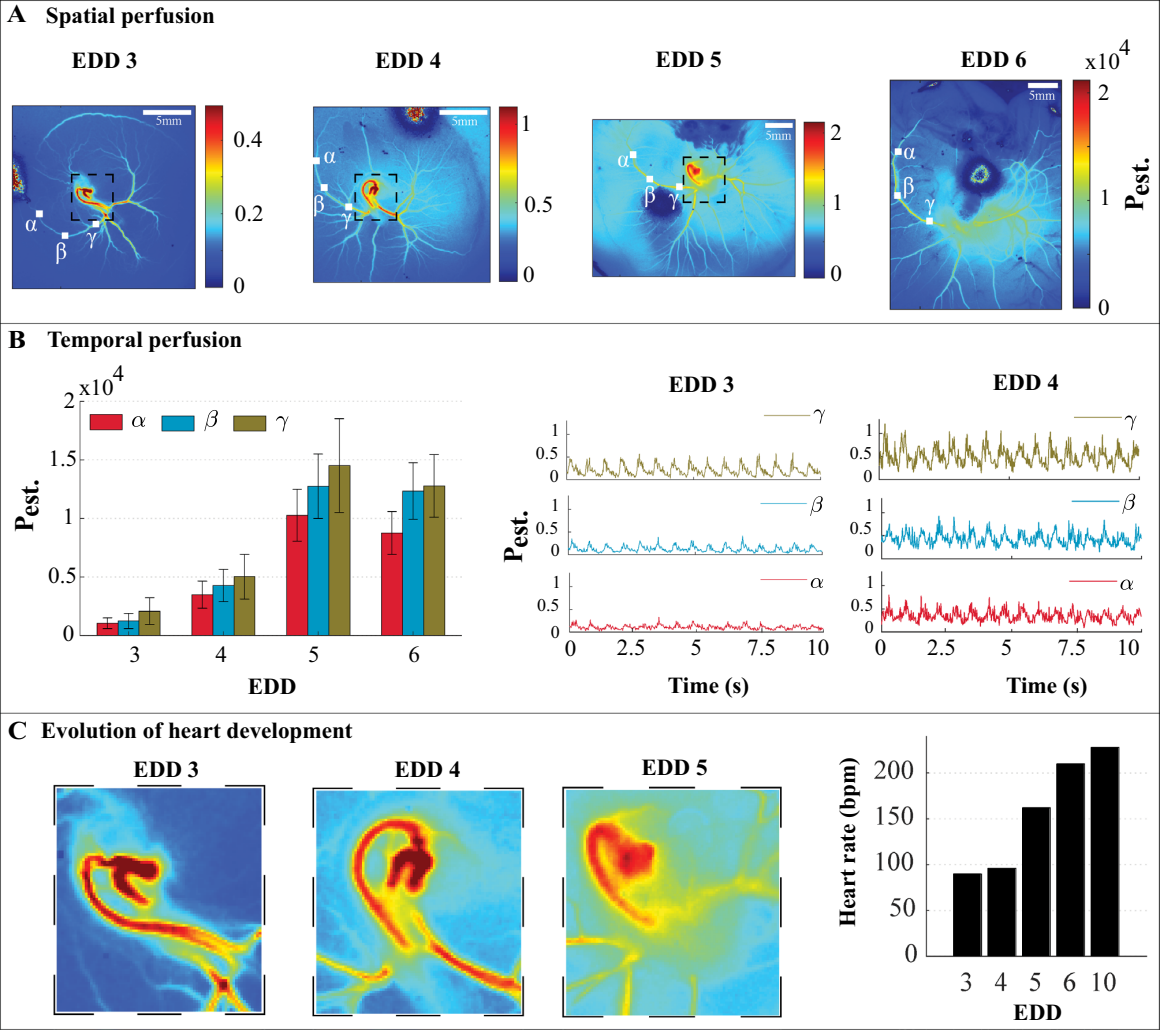
**Blood flow distribution of developing vascular networks.** Panel (**A**) shows the average spatial perfusion maps of complete vascular network of chick embryo belonging to developmental day 3 till 6. EDD, embryo development day. *P*_est._, estimated perfusion. Panel (**B**) shows the temporal perfusion profile from the regions in Panel (**A**) (highlighted using Greek symbols α = alpha, ß = beta and γ = gamma). Measured perfusion values are obtained from single independent biological sample (*n* = 1) over a timeframe of 10 s. Data of bar graph are mean ± standard deviation. Panel (**C**) represents zoomed snapshot of heart development from day 3 till 5 (highlighted regions in panel (**A**) using dashed boxes) and the heart rate of the sample. bpm, beats per minute.

The procedure of creating perfusion maps was described in Supplementary Information SI. Temporally averaged perfusion maps of developing chicken vasculature on days 3–6 are shown in Fig. 4(A). The spatiotemporal perfusion fluctuations within 10 s of recording on days 3–6 and 10 are visualized in Supplementary Video S8. One vessel of this sample is chosen for probing the perfusion fluctuations over developing days of 3–6. At each day, average temporal fluctuations at three locations of the selected vessel are calculated. Figure 4B shows an overview of the average temporal fluctuations over days as well as representative temporal fluctuations for days 3 and 4. Note that speckle contrast maps gathered on EDD10 with an exposure time of 10 ms were highly blurred due to the high blood flow within the samples combined with embryo movement artefacts. An alternative measurement with an exposure time of 5 ms was carried out which may preclude a comparison with EDD3-6. Therefore, this data is not included in the analysis of this subsection. More recently, it was shown that movement artefacts caused by a moving embryo can be corrected using an optical flow algorithm but that requires simultaneous RGB imaging using a color camera^31^.

For each EDD, the average estimated perfusion of the vessel decreases as the distance from the heart increases. This is due to the supply of blood to smaller vessels and capillaries on its way. An increase in the average estimated perfusion of all three regions from days 3 till 5 is also observed. The ratio of the average estimated perfusion on the region closest to the heart (*γ*) on day 5 compared to day 3 is calculated as 7. The underlying reason is the growth of the sample in terms of heart capacity, blood vessel numbers and generation of more blood. The heart shows the highest estimated perfusion level within the temporally averaged perfusion maps. In Fig. 4C the heart areas within days 3–5 are illustrated. The heart rate over developing days is calculated by counting the number of peaks in the temporal fluctuations of the estimated perfusion. For this sample, it increases from 90 beats per minute (bpm) on day 3 to 228 bpm on day 10.

#### Arterial-venous flow of developing vascular networks

**Figure 5:**
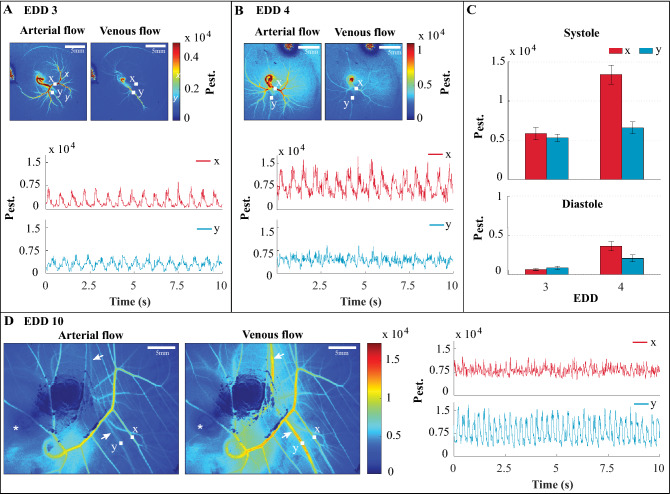
**Blood flow distribution within arteries and veins.** Panel (**A**), perfusion maps based on averaging of a number of frames chosen during systole (maximum perfusion) and diastole (minimum perfusion) from the region *x* that represent arterial and venous flow, respectively, for the development day 3. EDD, embryo development day. *P*_est_., estimated perfusion. Panel (**B**) represents the same analysis as Panel (**A**) but for the development day 4. Panel (**C**), a comparison between the averaged perfusion maps (mean ± standard deviation) for both systole and diastole during days 3 and 4 of the development. *x* and *y* correspond to the regions shown in the perfusion maps of Panels (**A**,**B**). Panel (**D**), perfusion maps and temporal fluctuations of the perfusion extracted from the regions *x* and *y* on the development day 10. The perfusion maps labeled with arterial and venous flow are calculated by averaging of a number of frames at minimum and maximum values of the perfusion at the region *y*, respectively. White arrows, the vein that provides a clear signal during the venous flow. Asterisk, the eye of the embryo. The exposure time for EDDs 3–4 is 10 ms while it is 5 ms for EDD 10.

As described in Supplementary Information SI, temporally averaged perfusion maps are created by the selection of perfusion maps at certain time points in order to visualize arterial and venous flow maps. For EDD3, a region x on a vessel close to heart (see Fig. 5A) is chosen to extract the temporal fluctuations of the estimated perfusion. Then, by choosing the frames correspond to maximum (systole) and minimum (diastole) values of region x, arterial and venous flow maps are formed. See Supplementary Video S9 that demonstrates the two flow maps in an overlapping manner. The arterial flow map shows the spread of the vessels supplied by the main arteries throughout the vascular network while the venous flow map shows high perfusion on the heart, a large vessel below it and the upper boundary of the vascular network. In Fig. 5B, the same analysis is performed for EDD4. The mean estimated perfusion on the regions x and y for the development days 3 and 4 are compared in Fig. 5C for both the systole and diastole cycles. For the systole cycle on region x, the mean estimated perfusion ratio of day 4 to 3 is 2.3, while this ratio is 1.2 on region y.

On EDD10, a fluctuation corresponding to the heartbeat pattern of the embryo is observed on the main vein labeled as region *y* (see Fig. 5D). The temporal fluctuations of the estimated perfusion on region x that corresponds to an arterial vessel is more stabilized than the region y. Here, the arterial and venous flow maps are formed by temporal averaging of the perfusion maps that belong to the minimum (diastole) and maximum (systole) points of the region y. The main vein labeled with the white arrows in Fig. 5D generates a higher signal during the venous flow. Based on white light imaging, this vessel has a darker red color compared to the arterial vessels which can be seen in Fig. 1C, left, indicating that the blood flowing through the vessel is less oxygenated and therefore venous. This observation can be explained as follows. The vessel labeled with y collects all the deoxygenated blood from the vasculature and directly reaches the heart, such that it moves slightly with each pulsatility of the heart (See Supplementary Video S6 EDD10). However, the vessel indicated with x is one of the many arterial branches that carry oxygenated blood. As a result, x consists of less blood flow and rhythmic pulsatility pattern compared with y.

### Erythrocytes visualization within individual blood vessels using SDF microscopy

**Figure 6:**
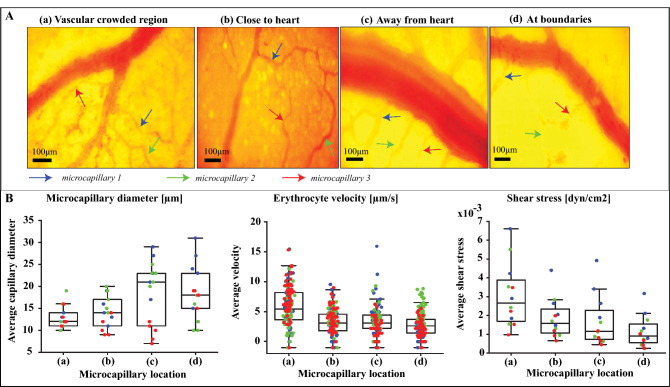
**Vessel diameter and erythrocytes velocity estimation within individual microcapillaries using SDF microscopy.** Panel (**A**) shows the time-averaged snapshots of microcapillaries organization at multiple locations of chick vascular networks. Three capillaries from each locations highlighted with colored arrows were selected for quantitative analysis. Panel (**B**) shows the quantitative analysis similar to Fig. 2B were performed on the selected microcapillaries from different locations to approximate the diameter and erythrocytes velocities. These values were served as an input for computational modeling to estimate the shear stress. For the shear stress profile, erythrocyte movements and tracking see Supplementary Figure S3, Supplementary Videos S10 and S11, respectively.

SDF microscopy as explained in Supplementary Information SI, was performed on multiple locations of the vascular network on EDD4. Figure 6A shows the temporally averaged images of different locations namely, vascular crowded region, close to heart, away from the heart and at the boundaries of the vascularized membrane. For videos of moving erythrocytes within multiple locations and their associated measurements see Supplementary Videos S10 and S11. The SDF images were used to elucidate the capillary structure as well as the movement of individual erythrocytes. Quantifications of microcapillary diameter and erythrocytes velocities are depicted in Fig. 6B. This information is further used as input for computational fluid dynamics simulations in order to predict fluid flow shear stresses in the capillaries. The results show an increasing trend in the microcapillary diameters starting from the heart towards the boundary regions of the vascularized membrane whereas the erythrocyte velocity flowing through these microcapillaries decreases since they are moving away from the heart. Interestingly, this is corroborated by the shear stress graph, which decreases on the regions from the heart to the boundaries. This points to an understanding that there is a decrease in blood flow resistance when the blood is supplied from heart to the surrounding tissues whereas it is the other way round when the blood is moving towards heart. Knowledge about shear stress is crucial for understanding the dynamics of the capillary network such as the formation of a new micro-vessel.

### Proof-of-concept study: LSCI on biofabricated tissue constructs

**Figure 7:**
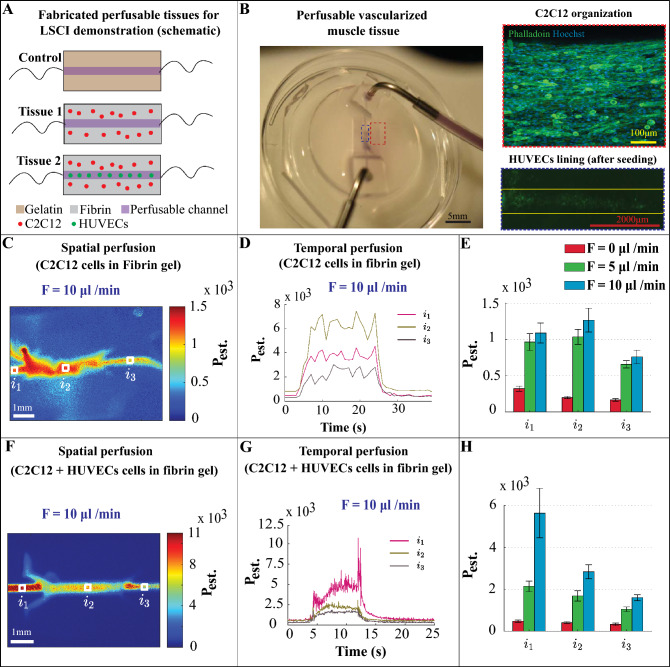
**LSCI on perfusable muscle tissues.** Panel (**A**) shows the schematic figure of three perfusable hydrogel tissue construct namely acellular gelatin hydrogel (control) , fibrin hydrogel with C2C12 cells (tissue 1) and fibrin hydrogel with C2C12 + HUVECs cells (tissue 2). Schematic figure were created using Adobe Illustrator. Data of gelatin hydrogel is illustrated in Supplementary Figure S5. Panel (**B**), left, shows an image of the perfusable muscle tissue construct with Intralipid-dye perfusion setup. Top right, a confocal microscope image of the C2C12 cellular organization and densities within tissue construct, highlighted with the dashed red box in the left image. Bottom right, a fluorescence microscope image of the channel area highlighted with the dashed blue box in the left image. Panel (**C**), illustrates the temporally averaged perfusion map (*ρ* = 0.85) of the flow phantom with C2C12 cells with a flow rate of *F* = 10 μl/min for the case of tube on Delrin. Panel (**D**), illustrates the temporal profiles of the estimated perfusion (*F* = 10 μl/min) associated with the white regions highlighted in Panel (**C**). Panel (**E**), illustrates the estimated perfusion for various flow rates. Panel (**F**) depicts the temporally averaged perfusion map (*ρ* = 0.85) of the flow phantom with C2C12 and HUVECs cells with the flow rate of *F* = 10 μl/min for the case of tube on Delrin. Panel (**G**), depicts the temporal profiles of the estimated perfusion (*F* = 10 μl/min) associated with white regions highlighted in Panel (**F**). Panel (**H**), depicts the estimated perfusion for various flow rates. Panels (**C**,**D**,**F**,**G**) are still images of Supplementary Video S12.

As described in methods section, the perfusable biofabricated tissue constructs were created and LSCI were performed using multiple flow rates. For the demonstrator experiment, we made three perfusable tissue constructs namely (1) acellular gelatin hydrogel (control), (2) fibrin hydrogel with C2C12 cells (tissue 1) and (3) fibrin hydrogel with C2C12 and HUVECs (tissue 2) as mentioned in Fig. 7A. The gelatin hydrogel was created for the initial demonstrator experiment. An image of the perfusable channel is shown in Supplementary Figure S5(A). The speckle contrast map for the flow rate of 28.5 μl/min is also shown in Supplementary Figure S5(B). The measured speckle contrast across the cross section of the channel for the case of no flow (Brownian motion) and the flow rate of 28.5 μl/min is depicted in Supplementary Figure S5(C). A plot of speckle contrast at the center of the channel versus the flow rate and the equivalent volumetric flux is depicted in Supplementary Figure S5(D). Here, we observe that increasing the flowrate, the measured speckle contrast is decreased. In this experiment, for the scattering ratio parameter (*ρ*) equals to 0.85, the estimated perfusion response to the set flow rate becomes linear. A plot of the estimated perfusion across the cross section of the channel for the case of no flow and the set flow rate of 28.5 μl/min is depicted in Supplementary Figure S5(E). Finally, the estimated perfusion versus the set flow rate and the corresponding volumetric flux is depicted in Supplementary Figure S5(F).

The representative image of a biofabricated channel and the corresponding confocal images for the case of C2C12 and HUVECs is depicted in Fig. 7B. The temporally averaged perfusion map for the channel with C2C12 cells with the set flow rate of 10 μl/min is illustrated in Fig. 7C. A temporal profile of the estimated perfusion at three locations, namely, inlet, middle and outlet for the set flow rate of 10 μl/min is shown in Fig. 7D. A comparison between the estimated perfusion at these three locations for the case of no flow and the set flow rates of 5 and 10 μl/min is illustrated in Fig. 7E. Similar analysis is performed for the channel with C2C12 and HUVECs cells and the data is shown in Fig. 7F–H. A video presentation of the flow application can be seen in Supplementary Video S12. To the best of our knowledge, this study is the first to show that laser speckle contrast imaging is compatible with bioengineered tissue constructs. By coupling fluid flow parameters to the organization of vascular networks, this leads to a deeper understanding regarding the structural information and mechanical environment of these developing vascular networks, which is an important component in controlling vascular organization in biofabricated tissues.

## Discussion

An artificial eggshell system was developed which enables the development of chick embryos up to embryo development day 10 and offers the possibility of studying the complete vascular network formation and organization over time. The culture system offers high accessibility as an area of 6 cm^2^ at the top of the system is open for optical probes. The culture system can accommodate 60 ml of fertilized egg contents (egg contents is measured to vary from 52 to 56 ml) and offers the flexibility for long-term chick embryo culture (Fig. 1). The secondary incubator (see Supplementary Figure S1) in which the samples were placed during imaging provided a relatively safe environment in terms of temperature and humidity control. On the other hand, we acknowledge that there are several challenges in the procedure of culturing samples and in the preparation for imaging, such as difficulties in cracking the eggshells at early development stage and transferring the egg content including the embryo into the artificial eggshell system.

To elucidate the relationship between vascular structure and organization, and shear stresses and perfusion values, multiple imaging modalities were used. The color imaging during the development days revealed global structural changes of the vasculature such as vessel area, vessel length and branching density (Fig. 2). The chick CAM vascular network includes large vessels and smaller capillaries that constantly change their diameter due to the remodeling mechanism^4^. Moreover, the fluid properties of fertilized egg contents such as thin/thick albumin and egg yolk change during the course of development. As this will result in a variation of the LSCI scattering profiles, we performed LSCI of flow phantoms consisting of microtubing of various diameters mounted on the light scattering and absorbing backgrounds. Dependency of the measured speckle contrast on both tube diameter and scattering/absorbing background was observed. A model that takes into account the scattering ratio was used to estimate perfusion. It is shown that with knowledge about the tube diameter and scattering level of the background, perfusion values can be estimated that are proportional to the actual volumetric flux or flow rate (Fig. 3). However, there are three reasons that prevent us to directly correlate flow phantom results to absolute blood flow values in the developing chick vasculature. (1) Although a blood mimicking fluid was pumped through microtubing in flow phantom experiments, it is different from the blood in terms of particle size and viscosity. (2) The scattering and absorbing backgrounds used in the flow phantom study were static and simplistic while the CAM model is a dynamic environment with its scattering and absorbing properties changing over developing stages. (3) In order to make the estimated perfusion values independent of tube diameters, vessel diameters in the CAM model must be accurately measured. Since there are blood vessels with various diameters at each development stage of the CAM model, blood vessel segmentation and characterization may be an intensive machine vision task that is out of the scope of this current research.

The tracking of spatiotemporal fluctuations of blood flow within vasculature of varying complexity and constantly changing vessel diameter, is important for validating the physiological significance of mechanical signals developed within the vascular networks. In this work, LSCI perfusion maps of the entire chick CAM vasculature within days 3–6 were obtained and the estimated perfusion levels on three locations of a vessel were compared over the development days (Fig. 4). It is known that distinct arteries and veins are formed within hours after the on-set of the heart beat in flow driven chick CAM vasculature^4^ . Earlier studies have shown that veins can transform into arteries when subjected to high shear stress forces by flow manipulation, proving that arterial-venous differentiation is a flow-driven highly dynamic process^4^. To our knowledge, this study is the first to show the blood perfusion within artery and veins separately over space and time at the developmental days 3–4 and 10 (Fig. 5).

In this study, SDF microscopy was performed in order to examine erythrocytes velocities in vessels that vary in size. With the analysis of the SDF data, microcapillary diameters and erythrocyte velocities were quantified. Then, with the combined data of velocity and diameter, fluid flow shear stresses for the microcapillaries of varying diameters were evaluated using computational modeling (Fig. 6B). This direct knowledge about shear stress is crucial for tissue engineers when perfusing biofabricated vascularized tissues with the goal of tuning the vascular organization, as fluid flow shear stress appears to be the main fluid flow related parameter that controls vascular organization^1,2^.

To illustrate that LSCI is not only compatible with developing chick embryos but also with bioengineered tissues, perfusable muscle tissue constructs were prepared using C2C12 mouse myoblast cells and GFP-HUVEC endothelial cells (Fig. 7). Even though the presented construct only contains a simple straight perfused channel, this study shows that LSCI imaging is compatible with engineered tissue constructs, thus highlighting the potential for the bioengineering community. Knowing the localized flow induced shear stress values and associated spatiotemporal information will help bioengineers to fine-tune the vascular organization using mechanical signals. We show that information about spatiotemporal perfusion can be obtained by LSCI in a non-invasive manner, using an affordable experimental setup and with a reasonable post-processing time. Recent studies show that intact-tissue imaging requires complicated staining procedure and expensive experimental setup for visualizing the structural and mechanical information^32^. This is in part due to the opaque nature of engineered tissue constructs. The tissue constructs that were used in this study were composed of gelatin and fibrin, which are commonly used hydrogel polymers. This provided us with different levels of opacity, in order to explore the LSCI application on engineered tissues. The addition of HUVECs to the fibrin hydrogel constructs decreases the vascular permeability which is visible during perfusion (See Supplementary Video S12). To our knowledge, this study is the first to show that LSCI is compatible with engineered tissues and that LSCI can aid in the study of endothelial barrier properties. Future work will focus on perturbing the developing chick embryo using external localized fluid flows and multiple growth factors. Additionally, the chick embryo culture system as well as the perfusable engineered muscle tissue model will be improved to further elucidate the role that fluid flow related mechanical signals can play in the tuning of vascular organization.

## Methods

### Fabrication of *ex ovo* culture system

The culture system was made of polydimethylsiloxane, PDMS (Sylgard 184 silicone elastomer, Dow corning). The PDMS base was mixed at 10:1 (w/w) with the curing agent and poured into a laser cut circular shaped polymethyl methacrylate (PMMA) block . The mold was placed in a desiccator to remove the air bubbles, and it was cured overnight in a 65 ºC dry oven. Then, the cured PDMS and PMMA mold was carefully detached using a sharp razor blade.

### Culturing of chick embryos

Fertilized chicken eggs (Dekalb white) were purchased from Het Anker bv (Ochten, The Netherlands) and stored at a temperature of 12 ºC. 24 h prior to the egg transfer, the modified incubator was turned on and maintained at 38 °C and 65% humidity throughout the entire culturing process (see Supplementary Figure S1). For the first 3 days of incubation, the eggs were turned for 15 s every two hours to make sure that the embryo does not stick to the wall of the egg shell. After 3-day incubation period, the chicken embryo was transferred to the PDMS culture system in a sterile environment. As a first step, in order to avoid damage to the yolk and embryo vasculature, using sharp tweezers a hole of about ~ 2 mm diameter was created on the eggshell and by inserting an 18G syringe microneedle 3 ml of albumin was withdrawn using a plastic syringe. The removed albumin was carefully added to the culture system while limiting the introduction of air bubbles, after which the egg content was transferred.

### Measurement of vascular network properties

The color images of the chick vascular networks were acquired at the indicated development days as explained in methods section, and the vascular network information such as vessel area, average vessel diameter, average vessel length, number of branching points or junctions and mean lacunarity were measured using ImageJ (Fiji) software and Angiotool plugin^33^. The image analysis pipeline is described in Supplementary Figure S2. Graphs were plotted using OriginPro 2019b.

### Computational modeling and fluid dynamics simulations

Computational models were used to calculate the fluid flow shear stresses of the developing vascular networks (for detailed information, see Supplementary Figure S3). Structural information such as microcapillary diameter calculated from SDF microscopy served as the geometrical input of the simulation model. Straight tubes that mimic blood vessels of varying diameter of 7–31 µm (see Fig. 6A for the selected microcapillaries, highlighted with blue, green and red arrows) were designed and flow studies were performed using COMSOL Multiphysics software version 5.5. Boundary conditions include input flow velocities obtained from the SDF microscopy videos (for erythrocyte tracking, see Supplementary Video S11 and Fig. 6B for erythrocyte velocities) and zero pressure conditions at both the input and output were used. For this study, blood was assumed as a Newtonian fluid with the density 1060 kg/m^3^ and dynamic viscosity 10^–4^ Pa.s. Even though blood is in essence a non-Newtonian liquid, the non-Newtonian properties of chicken blood were shown to be limited compared to human blood^34^. Flow type was considered as laminar and a vessel length of 1 mm was used.

### Biofabricated perfusable muscle tissue constructs

Muscle tissue perfusion chambers (see Fig. 7) were replica molded using PDMS, similar to the fabrication of eggshell culture system. The dimensions of the chambers were 8 mm in length, 5 mm in width and 2 mm in depth. Culture chambers were supplied with nylon wires (0.5 mm diameter) before the chambers were filled with hydrogel.

Mouse myogenic C2C12 cells were obtained from ATCC and cells were used up until passage number 21. Myoblasts were cultured in Dulbecco’s modified Eagle’s medium (DMEM) supplemented with 10% fetal bovine serum and 1% penicillin–streptomycin. Green fluorescent protein (GFP) expressing human umbilical vein endothelial cells (HUVECs) were obtained from Cellworks and cells were used up until passage number 5. GFP-HUVECs were cultured in EGM-2 medium (EGM-2 Basal medium + EGM-2 Supplements) with 1% (v/v) Pen/Strep. All cells were maintained in a humidified incubator kept at 37 °C and 5% CO2 and were passaged upon reaching 80% confluency.

A-cellular controls were prepared using gelatin. 5% gelatin hydrogel solution was prepared by mixing Type A 300 bloom porcine skin gelatin (G1890-500G, Sigma Aldrich) with demineralized water and stirred at 200 rpm at 65ºC for 30 min. Fibrin hydrogel was prepared by mixing 0.5 U/ml, 1 mg/ml and 6 mg/ml of thrombin, CaCl_2_ and fibrinogen respectively. C2C12 cells were resuspended in culture medium and were then mixed with fibrin hydrogel to reach a final cell concentration of 1.5 × 10^7^ cells/ml and a final fibrinogen concentration of 6 mg/ml. 100 μl of this mixture was then transferred to the perfusion chamber. After gelification, culture medium was added and the samples were incubated at 37 °C and 5% CO_2_.

The nylon wires were removed after 7 days, resulting in a perfusable channel. The channels of select samples were seeded with 3.5 × 10^7^ cells/ml of GFP-HUVECs for 24 h. LSCI was performed on day 8.

### Ethics statement

According to the Dutch animal care guidelines, IACUC approval for chicken embryo experimentation is not necessary unless hatching is expected. Moreover, only experiments with chick embryos of development EDD14 and older need IACUC approval. The embryos used in this study were all in early stages of embryo development (between EDD3 and EDD10).Fertilized chicken eggs used in this study were purchased from approved poultry egg farms in Netherlands. GFP-HUVECs culture studies were approved by Dutch Ministry and experiments were performed under ML-1 laboratory environment, followed by the university policies and standard protocol.

## Supplementary Information

Supplementary Information S1.

Supplementary Video S6.

Supplementary Video S7.

Supplementary Video S8.

Supplementary Video S9.

Supplementary Video S10.

Supplementary Video S11.

Supplementary Video S12.

## Data Availability

The datasets generated during and/or analyzed during this study to reproduce Fig. 1(A), top; Fig. 1(C); Fig. 2 raw RGB images; Fig. 3(C-H); Figs. 4-5; Fig. 6(A); Fig. 7(C-H); Supplementary Fig. S4 and Supplementary Fig. S5(B-F) are available in the Figshare repository 10.6084/m9.figshare.16402662. The rest of the datasets generated during and/or analyzed during this study to reproduce Fig. 2(B); Fig. 6(B) and Supplementary Figs. S2-S3 are available from the corresponding author on reasonable request.
