# Supplementary material for: Assessment of flow within developing chicken vasculature and biofabricated vascularized tissues using multimodal imaging techniques
Source: Sci Rep. 2021 Sep 14;11:18251. doi: 10.1038/s41598-021-97008-w (PMC8440514; doi:10.1038/s41598-021-97008-w)
Supplement: Supplementary file 1 — Supplementary Information S1. [file 41598_2021_97008_MOESM1_ESM.pdf]

Supplementary Information SI

Assessment of flow within developing chicken vasculature and biofabricated vascularized tissues using multimodal imaging techniques

Prasanna Padmanaban[1,ⱡ], Ata Chizari[2,ⱡ], Tom Knop[2], Jiena Zhang[1], Vasileios D. Trikalitis[1], Bart Koopman[1], Wiendelt Steenbergen[2,*], and Jeroen Rouwkema[1,*]

[1]Vascularization Lab, Department of Biomechanical Engineering, Technical Medical Centre, Faculty of Engineering Technology, University of Twente, 7500 AE Enschede, The Netherlands

[2]Biomedical Photonic Imaging, Technical Medical Centre, Faculty of Science and Technology, University of Twente, 7500 AE Enschede, The Netherlands

ⱡthese authors contributed equally to this work
*corresponding author: j.rouwkema@utwente.nl and w.steenbergen@utwente.nl

Color imaging and laser speckle contrast imaging of chick vasculature experiments

For colour imaging, samples were illuminated using light emitting diodes (TYPb0913 Jansjö LED). A color camera (Basler acA1920-40ucUSB3) was used for RGB imaging at full frame with the rate of 30 Hz and exposure time of T=10 ms. To realize an image resolution of up to 17.9 micron/pixel a 25 mm camera objective (Pentax TV lens) was used. For a wider FOV and realizing image resolution of up to 55 micron/pixel, a 12 mm camera objective (FUJINON HF12XA-5M) was used. The maximum FOV for color imaging was 71.7×73.1 mm^2.

For LSCI, a continuous wave and coherent single longitudinal mode laser (CNI MSL-FN-671) with a wavelength of λ=671 nm was used. The laser light was focused on a single mode optical fiber (Thorlabs P1-630A-FC-5) using an Olympus plan achromatic microscope objective (Thorlabs, RMS10X). A 20 degrees top hat square engineered diffuser (Thorlabs ED1-S20-MD) was located at the distal end of the optical fibre. The laser power illuminated from the optical fibre to the engineered diffuse was around 20 mW. A monochrome camera (Basler acA2040 55um) recorded raw speckle frames with an exposure time of T=10 ms, frames rate of 50 Hz, gain of 0 dB and acquisition time of 10 seconds. The sample at development day of 10 was imaged with both T=5 ms and T=10 ms. Using a 25 mm camera objective (Pentax TV lens) with an effective numerical aperture of F8, a spatial resolution of up to 15.4 micron/pixel and a maximum FOV of 37×38.4 mm^2 was achieved. To suppress the background light, a hard coated bandpass interference filter of wavelength 675±12.5 nm (Edmund Optics) was placed in front of the monochrome camera objective. To suppress the effect of specular reflection from the laser light, a linear polarizer of wavelengths 600-1100 nm (Thorlabs LPNIRE100-B) with a direction perpendicular to the laser light polarization was mounted in front of the camera objective.

Each speckle frame was converted to a contrast map by applying a sliding window of size 7×7 pixels using sliding convolution technique. The equation for speckle contrast is[1]

$$C = \frac{\sigma_I}{\bar{I}} \qquad (1)$$

where $\bar{I}$ and σ_I are mean image value and image value fluctuations about the mean image value in the region of interest (ROI), respectively. With the assumption of a negative exponential form for the field autocorrelation function, the speckle contrast can be written as [2,3]

$$C = \sqrt{\left(\frac{\tau_c}{T} + \frac{\tau_c^2}{2T^2}\left[\exp\left(-\frac{2T}{\tau_c}\right) - 1\right]\right)} \qquad (2)$$

where τ_c and T are correlation time and exposure time, respectively. The correlation time is assumed to be[4]

$$\tau_c = \frac{\alpha}{v_c} = \frac{1}{P} \qquad (3)$$

where v_c is the effective velocity and is called perfusion in arbitrary units in our study. α is a constant and is set 1 in this study; thus, $v_c = P$. Based on the set exposure time (T), a lookup table is made to assign a perfusion value ($P_{est.}$) to each measured speckle contrast ($C_{meas.}$) using linear interpolation. For the perfusion maps of **Figure 4(A)** at each measurement, all frames were averaged to make a representative graph. For the perfusion maps of **Figure 5** at each measurement, 10 % of the captured frames were selected for the averaging based on the maximum or minimum perfusion values at the ROI. The set colour bar scaling for each experiment shown in **Figure 4(A)** and **Figure 5** corresponds to mean+7 times the standard deviation of the entire temporally averaged perfusion map. The data analysis for perfusion imaging is done in MATLAB R2019b.

Laser speckle contrast imaging of microtubing flow phantoms

The laser illumination was as described in above-mentioned section with the difference that the diffuser was removed in order to provide higher intensity level. The CMOS camera type and its setting was as above mentioned. Here, instead of a camera objective, a 4-*f* imaging system of magnification $M \approx 1$ (image resolution of 3.3 micron/pixel) was built using two biconvex lenses (Thorlabs f=75 mm) and a diaphragm located at the focal point between the two lenses. The diaphragm was adjusted to make speckle size of approximately 3×3 pixels on the camera sensor.

Table S1: Types of microtubing channels and the corresponding micro-syringes

Channel No.	Microtubing type	Microtubing inner diameter [µm]	Microtubing outer diameter [µm]	Micro-syringe type	Micro-syringe volume [µl]
1	HPFA, Upchurch Scientific 1931	75	360	ILS, 2100506	25
2	HPFA, Upchurch Scientific 1932	100	360	ILS, 2100606	50
3	HPFA, Upchurch Scientific 1933	150	360	ILS, 2100706	100
4	FEP, Upchurch Scientific 1688	200	800	ILS, 2100706	100
5	PFA, Scientific Commodities BB310-30	300	635	Norm-Ject, Luer	1000
6	Autoclavable Nylon Tubing, LOT	500	630	Norm-Ject, Luer	1000

In order to mimic the flow within the hollow structures of blood vessels, microtube phantoms were used. This includes the use of dialyses tubes[5] along with the 3D printed tubes made up of soft materials[6]. In

this study, six pairs of tubing channels were coupled to syringes (see **Table S1** that also specifies the tube materials). The tubing channels were located on a static scattering object called Delrin and on a black tape (black anodized sheet). A wall was located between the Delrin plate and the black taping area to prevent light leakage (see **Figure 3(A)**). To suppress the possible lens effect created by the wall curvature of tubing being imaged on the camera, microscope glass slides of 1 mm thickness (Thermo Scientific, Menzel-Glaser) were mounted on the tubing channels to form an enclosed space which was filled with ultrasound gel (Sonogel, Vertriebs GMBH). For creating a blood-resembling fluid in terms of optical properties at the wavelength of λ=671 nm, a stock solution of 830 µl Intralipid 20 % (Fresenius Kabi Nederland BV) with reduced scattering coefficient[7] of 26 mm^{-1} and 43 µl Ecoline 700 ink (Talens) with the measured absorption coefficient of 24.6 mm^{-1} and 10 ml demineralized water was prepared. The volume fractions were chosen such that the prepared solution had a reduced scattering of 2 mm^{-1} and absorption coefficient of 0.1mm^{-1} to mimic the scattering and absorption properties of blood at a wavelength of λ=671 nm[8]. For each tube diameter, the coupled syringe was installed in a pump (Aladin-1000). The average flow speed (volumetric flux) through the tubing was calculated as

$$V = \frac{F}{A} \tag{4}$$

where F and A stand for flow rate and tubing area, respectively. Therefore, the dimension of V is unit volume per unit time per unit area or simply unit distance per unit time. A fluid speed range of 0 to 7.5 mm/s was applied for each experiment in 10 steps during which 250 raw speckle frames were recorded. **Table S2** shows the set flow rates for each tube diameter.

To account for the effect of static scattering caused by any static scatterer contributing to the observed dynamic speckle, the speckle contrast has the form[9]

$$C = \sqrt{\beta\left[\rho^2\frac{\exp(-2x)-1+2x}{2x^2} + 4\rho(1-\rho)\frac{\exp(-x)-1+x}{x^2} + (1-\rho)^2\right]} + C_{noise} \tag{5}$$

where x=T/τ_c and β is a constant that may be used for calibration. The scattering ratio parameter $\rho = \frac{I_f}{I_f+I_s}$ where I_f is the time-averaged intensity of fluctuating dynamic scattered light and I_s the intensity of the statically scattered light. For ρ=1 (i.e. fully dynamic scattering), Eq. (5) will be equal to Eq. (2) provided that β=1 and C_{noise}=0. Thus, for the sake of consistency, we set β=1 and C_{noise}=0. For each tube diameter a scattering ratio parameter is chosen such that a linear response of the perfusion values to the applied flow rates is achieved (see **Table S3**).

Table S2: Flow rates for various tube diameters to obtain the same speed interval

			Calculated speed [mm/s]									
			0	0.5	1	2	3	4	5	6	7	7.5
Microtubing inner diameter [µm]	75		0	0.1	0.3	0.5	0.8	1.1	1.3	1.6	1.9	2
	100		0	0.2	0.5	0.9	1.4	1.9	2.4	2.8	3.3	3.5
	150		0	0.5	1.1	2.1	3.2	4.2	5.3	6.4	7.4	8
	200		0	0.9	1.9	3.8	5.7	7.5	9.4	11.3	13.2	14.1
	300		0	2.1	4.2	8.5	12.7	17	21.2	25.5	29.7	31
	500		0	5.9	11.8	23.6	35.3	47.1	58.9	70.7	82.5	88.4
			Set flow rate [µl/min]									

Table S3: Scattering ratio parameters set for various microtubing diameters scattering and absorbing background types

Tube on Delrin	Microtubing inner diameter [µm]	75	100	150	200	300	500

Tube on black	ρ	0.31	0.31	0.32	0.42	0.7	0.87
	ρ	0.47	0.47	0.63	0.71	0.84	0.9

To obtain a fit for the measured speckle contrast versus perfusion with the Eqs. (3) and (5) for each tube diameter, speckle contrast from Eq. (5) was plotted versus a shifted and scaled version of the effective velocity of Eq. (3) as

$$v_c' = av_c + b \tag{6}$$

The shifting and scaling parameters where chosen based on a visual fit with the measured data and the goodness of this fitting was examined by the R^2 value of a linear fitting of the estimated perfusion versus the actual fluid velocity (volumetric flux). See **Table S4** for an overview of the fitting factors for the entire flow phantom experiments.

Table S4: List of fitting factors for theoretical fitting of the flow phantom experiments

	Microtubing inner diameter [μm]	75	100	150	200	300	500
Tube on Delrin	a	70	120	340	550	990	2500
	b	220	220	-100	-300	-750	-2200
	R^2	0.89	0.86	0.67	0.88	0.99	0.99
Tube on black	a	80	170	320	650	850	2000
	b	-80	-100	-300	-450	-450	-1000
	R^2	0.95	0.90	0.96	0.91	0.96	0.97

Side-stream dark field microscopy

SDF uses LEDs (with a center wavelength of 530 nm) as light source, present at the probe tip that directly penetrate deep into the specimen[10]. This wavelength corresponds to an isosbestic point in the absorption spectra of both deoxy- and oxyhemoglobin. The high absorption at this wavelength causes moving RBCs to appear black against a gray background in the SDF raw data. In order to give them a natural look we used a false color map ranging from red (associated with RBCs) to yellow (associated with the yolk). In SDF, the imaging system is surrounded by the light sources in a coaxial geometry. The dark field imaging arrangement avoids direct surface reflections. This way, clear images of the capillaries were captured without blurring. Due to low battery power consumption and portability, this technique can be used in different experimental scenarios.

A microscan video microscope system (Microscan Microvision Medical) was used for SDF microscopy with a spatial resolution of 1.4 micron/pixel. The video microscope probe was mounted on a vertical motorized stage (Zaber X-LHM200A-E03). The probe was gently moved toward the CAM vasculature until it touched the desired region. Then, the frames were acquired with a rate of 30 Hz for 12 seconds. The representative SDF image for each measurement was an average of 25 consecutive frames.

References

1. Freund, I. Joseph W. Goodman: Speckle Phenomena in Optics: Theory and Applications. *J. Stat. Phys.* **130**, 413–414 (2007).
2. Fercher, A. F. & Briers, J. D. Flow visualization by means of single-exposure speckle photography. *Opt. Commun.* **37**, 326–330 (1981).
3. Duncan, D. D. & Kirkpatrick, S. J. Can laser speckle flowmetry be made a quantitative tool? *J. Opt. Soc. Am. A* **25**, 2088 (2008).
4. Briers, J. D. and W. S. Laser speckle contrast analysis (LASCA): A Nonscanning, full-filed technique for monitoring capillary blood flow. **1(2)**, 174–179 (1996).
5. Christensen-Jeffries, K. *et al.* 3-D in Vitro Acoustic Super-Resolution and Super-Resolved Velocity Mapping Using Microbubbles. *IEEE Trans. Ultrason. Ferroelectr. Freq. Control* **64**, 1478–1486 (2017).

6. Ommen, M. L. *et al.* 3D printed calibration micro-phantoms for super-resolution ultrasound imaging validation. *Ultrasonics* **114**, (2021).
7. Michels, R., Foschum, F. & Kienle, A. Optical properties of fat emulsions. *Opt. Express* **16**, 5907 (2008).
8. Meinke, M., Müller, G., Helfmann, J. & Friebel, M. Optical properties of platelets and blood plasma and their influence on the optical behavior of whole blood in the visible to near infrared wavelength range. *J. Biomed. Opt.* **12**, 014024 (2007).
9. Boas, D.A. & Dunn, A.K. Laser speckle contrast imaging in biomedical optics. J. Biomed. Opt. 15, 011109 (2010).
10. Goedhart, P. T., Khalilzada, M., Bezemer, R., Merza, J. & Ince, C. Sidestream Dark Field (SDF) imaging: a novel stroboscopic LED ring-based imaging modality for clinical assessment of the microcirculation. *Opt. Express* **15**, 15101 (2007).

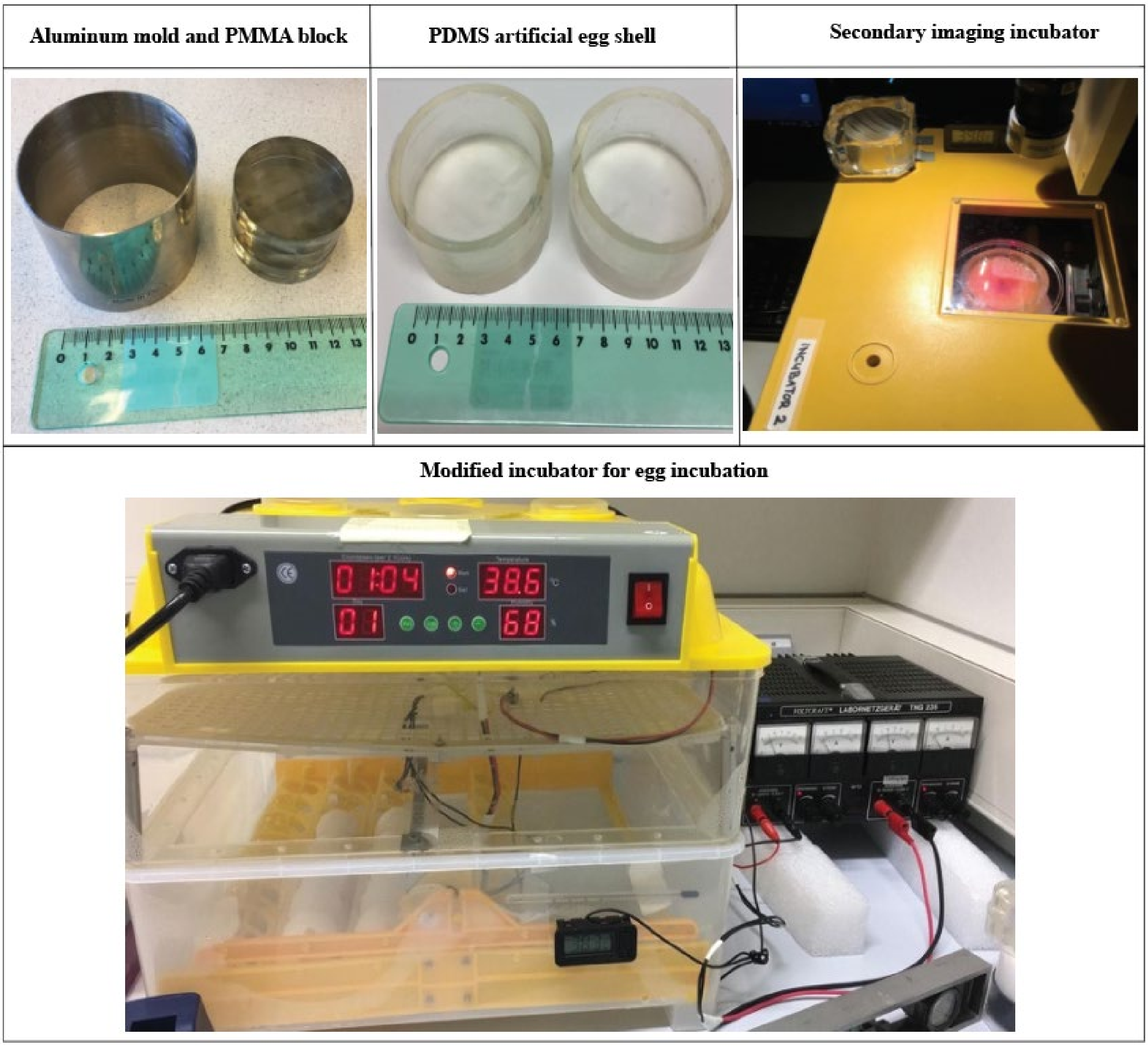

Supplementary Figure S1. Complementary data for artificial eggshell fabrication and fertilized chicken eggs incubation Top panel (left and middle) shows the aluminum mold and circular shaped laser cutted PMMA block, used for PDMS casting to make the artificial eggshell, where fertilized chicken embryos are cultured. **Top panel** (right), secondary incubator with artificial eggshell cultured chick embryo sample used for multimode imaging experiments. **Bottom panel,** shows the modified egg incubator with controllable fan and additional temperature sensors. Fertilized chicken eggs and artificial eggshell cultured chick embryo samples were incubated in this incubator throughout the study. Only during the imaging, artificial eggshell cultured chick embryo samples were transferred to the secondary imaging incubator.

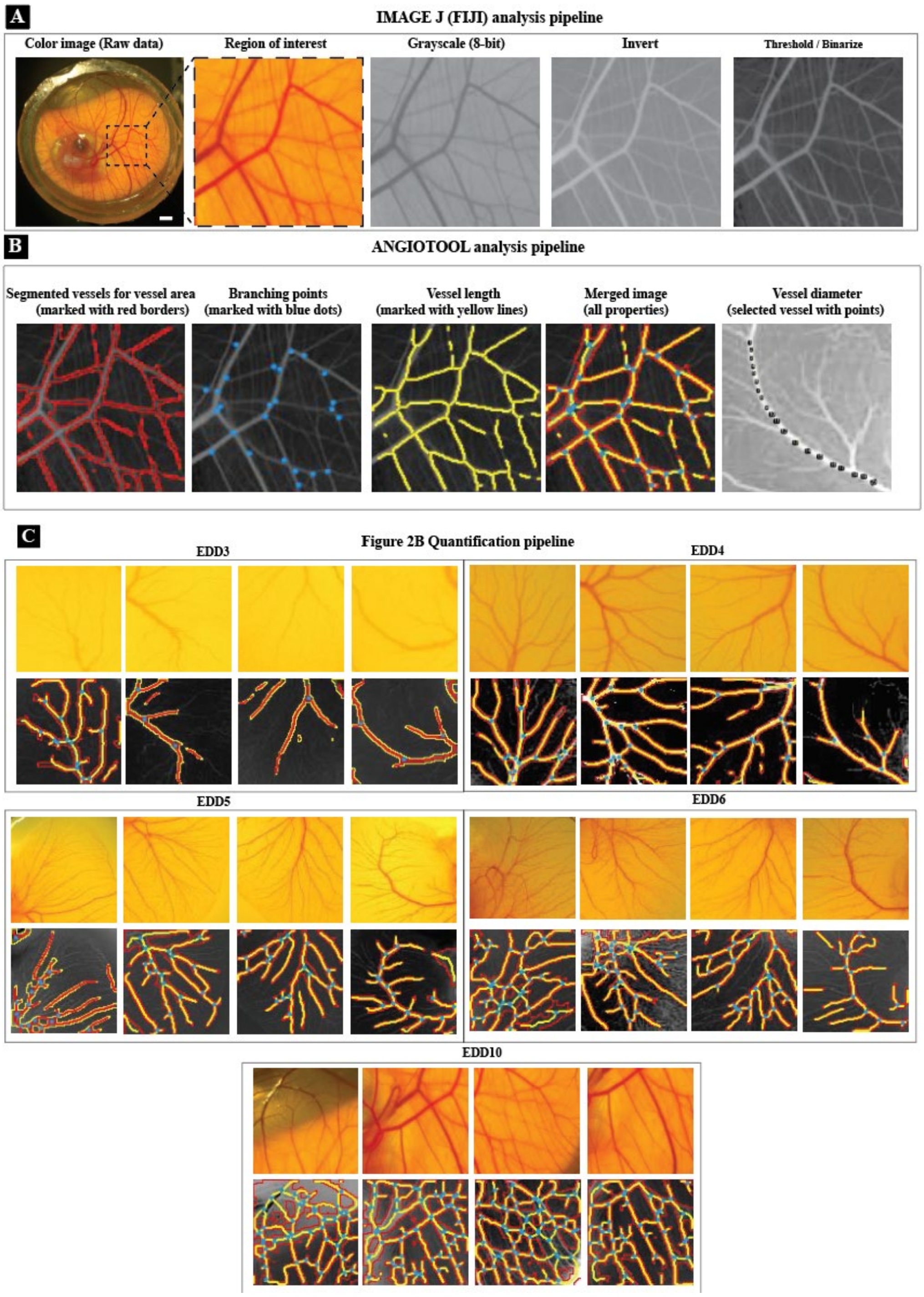

Supplementary Figure S2. Complementary data for quantitative image analysis of vascular networks. **Panel A** shows the steps involved in binarizing of RAW data using ImageJ (FIJI) software tool for vessel diameter and length measurement. **Panel B** shows the steps involved in Angiotool Plugin, used for calculating vascular network properties such as vessel length, vessel diameter, branching points and lacunarity. **Panel C** shows the colored raw data of selected ROIs and respective merged images of vessel properties obtained from Angiotool Plugin. These measured values are used for plotting graphs in **Figure 2(B)**.

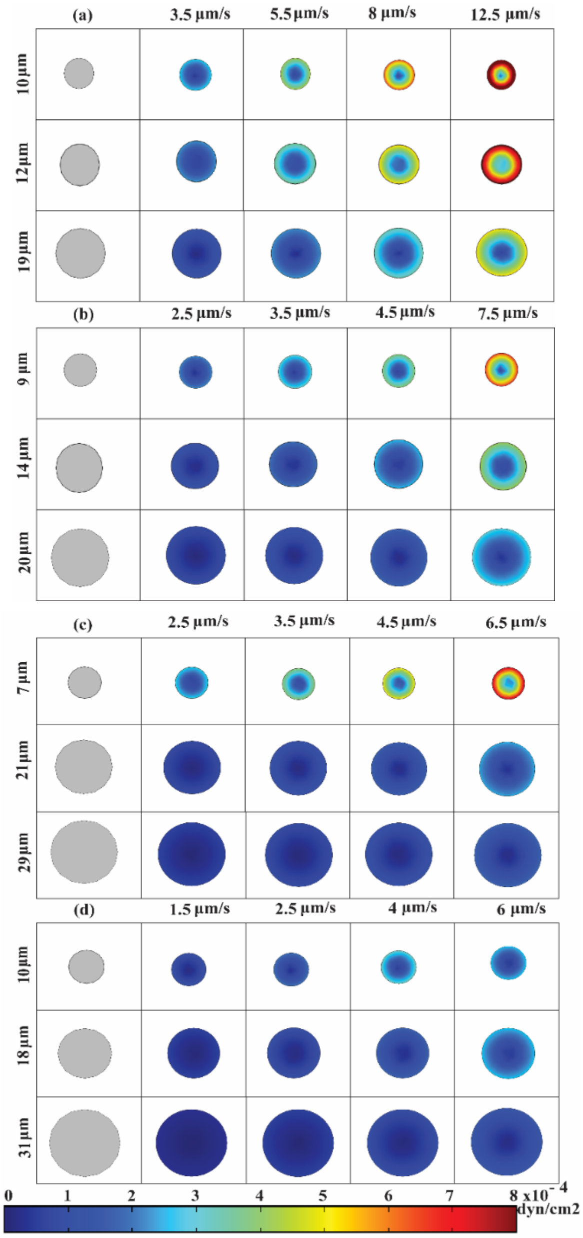

Supplementary Figure S3. Computational models showing the average shear stress values within varying microcapillary diameters obtained from SDF microscopy data.

Panels (a-d) shows the cross-sectional view of selected microcapillary diameters from SDF microscopy data, which are highlighted in **Figure 6(A)**. By coupling the microcapillary diameters and erythrocyte velocities from SDF microscopy data, the average volumetric shear stresses are computed for different velocities using COMSOL Multiphysics software Version 5.5 as shown in the panels (a-d). Three microcapillary from four different regions are selected for the diameter estimation and erythrocyte tracking. Selected capillaries are highlighted in blue, green and red arrows (refer **Figure 6(A)**). Min, Mid and Max values of microcapillary diameters are taken from **Figure 6(B)** (left graph), used as structural input geometry for the computational models. 25, 50, 75% and max values of erythrocyte velocities are taken from **Figure 6(B)** (middle graph), as input velocities. In total combination of 12 microcapillary geometries with varying diameters and 16 different input velocities were tested. Refer **Figure 6(B)** for average shear stress graph. See **Supplementary Videos S10** and **S11** for erythrocyte movements and tracking.

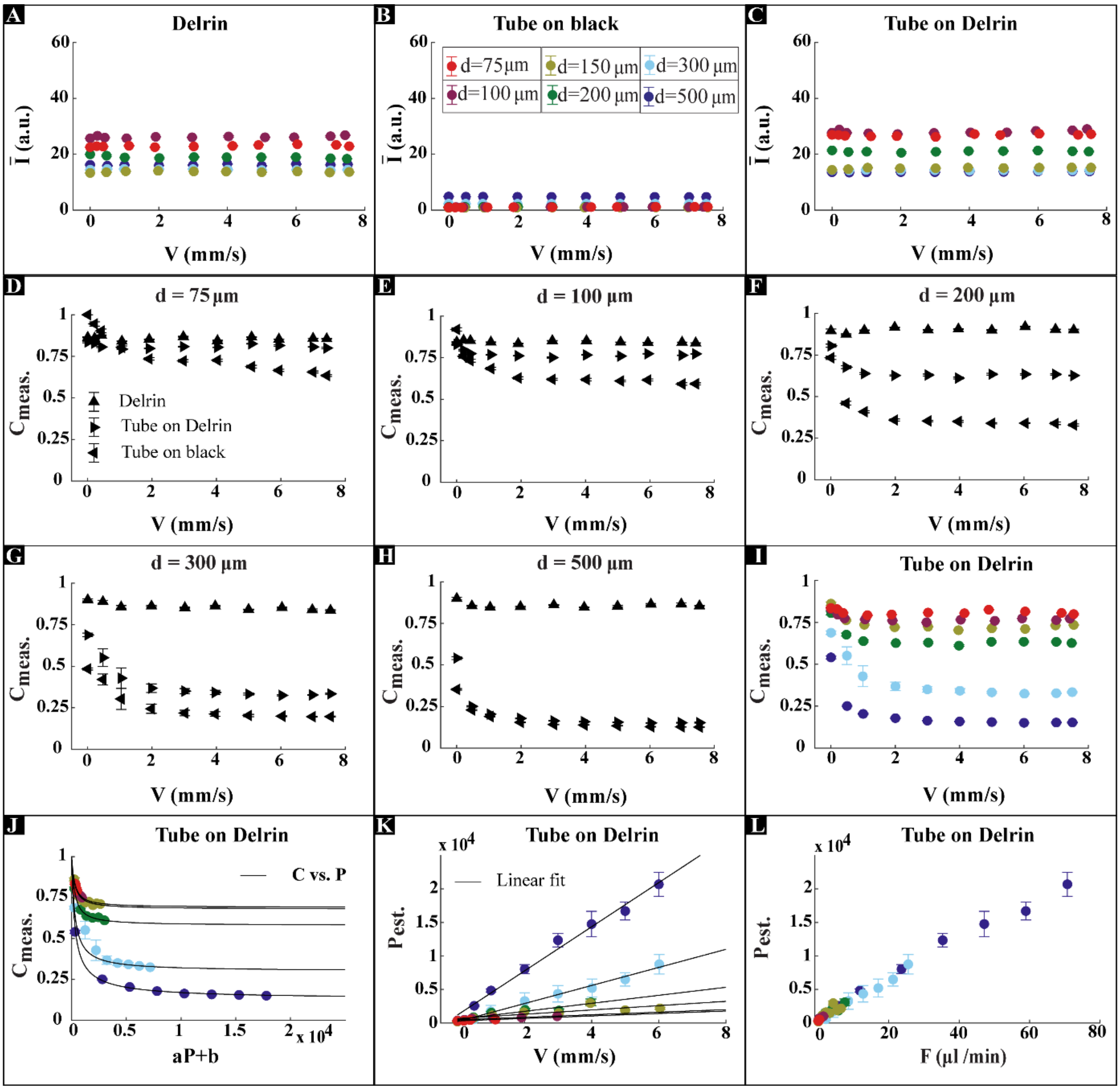

Supplementary Figure S4. Complementary data of LSCI for microtubing flow phantoms.
Panels A-C, mean intensity versus the volumetric flux for Delrin, tube on black and tube on Delrin, respectively. Data points are mean ± standard deviation. The colored data points in **Panels A-C, I-L** correspond to various tube diameters which are defined in **Panel B**. **Panels D-H,** measured speckle contrast versus the volumetric flux as a comparison of Delrin, tube on Delrin and tube on black for tubing diameters of 75, 100, 200, 300 and 500 µm, respectively. **Panels I-L** correspond to the case of tube on Delrin. **Panel I,** measured speckle contrast versus the volumetric flux. **Panel J,** measured speckle contrast versus the shifted and scaled versions of perfusion overlapped with theoretical relation between the speckle contrast and perfusion. **Panel K,** estimated perfusion versus the applied volumetric flux with a linear fit for each tube diameter. **Panel L,** estimated perfusion versus the flow rate.

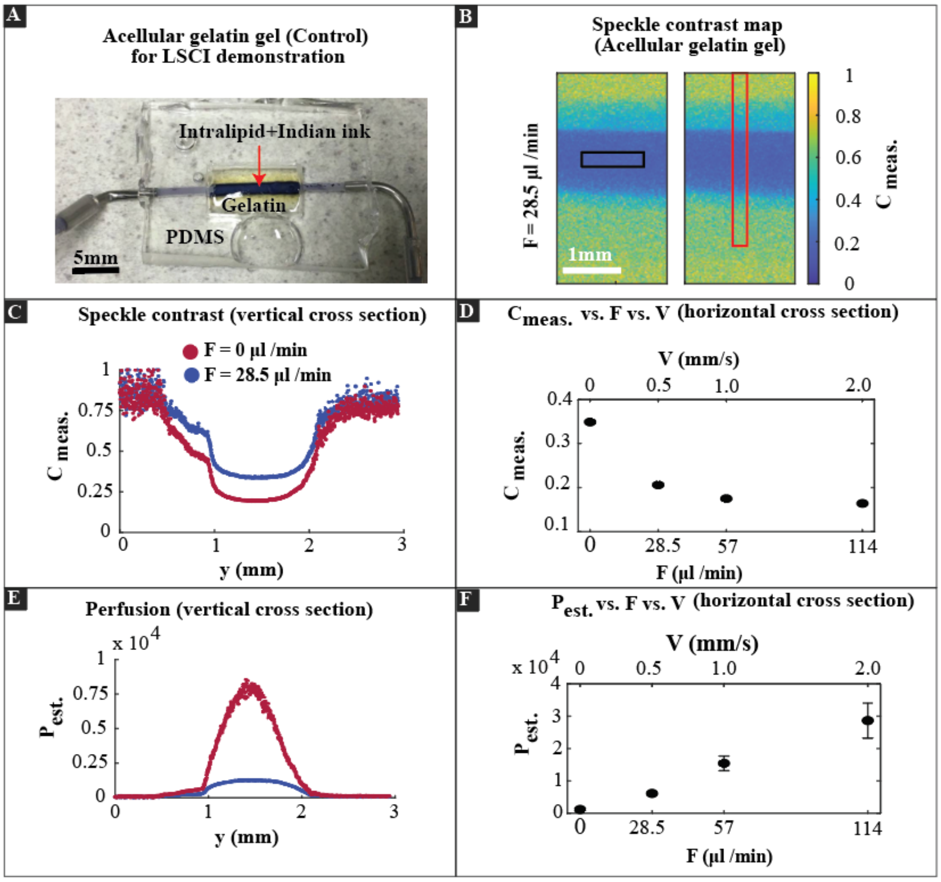

Supplementary Figure S5. Complementary data of LSCI flow phantom on gelatin hydrogel.
Panel A shows the perfusable gelatin hydrogel construct without cells. Gelatin hydrogel was used for initial demonstrator testing and it also served as control and substitute to plain fibrin hydrogel construct. **Panel B** shows the spatial speckle contrast map for the case of tube on black. **Panel C** shows speckle contrast over the cross sectional vertical region (highlighted with red box) indicated in **Panel B**. **Panel D** shows speckle contrast versus flow rate and volumetric flux calculated from the horizontal region (highlighted with black box) indicated in **Panel B**. **Panel E** depicts the estimated perfusion (ρ=0.85) associated with the speckle contrast shown in **Panel C. Panel F** depicts the estimated perfusion (ρ=0.85) associated with the speckle contrast shown in **Panel D**.

Supplementary Videos

Supplementary Video S6. Videos of chick embryo movement and heart beat

Supplementary Video S7. Microtubing flow phantom

Supplementary Video S8. Spatial and temporal perfusion profile videos of developing vascular networks

Supplementary Video S9. Toggled video – artery and vein

Supplementary Video S10. Moving erythrocytes within individual capillaries at multiple locations

Supplementary Video S11. Erythrocyte tracking

Supplementary Video S12. LSCI on biofabricated perfusable muscle tissues
